# Cardiometabolic risk factors predict executive function scores in high-risk individuals

**DOI:** 10.1093/geroni/igab046.2454

**Published:** 2021-12-17

**Authors:** Joshua Gills, Megan Jones, Anthony Campitelli, Sally Paulson, Erica Madero, Jennifer Myers, Jordan Glenn, Michelle Gray

**Affiliations:** 1 University of Arkansas at Fayetteville, Fayetteville, Arkansas, United States; 2 University of Arkansas at Fayetteville, Cincinnati, Ohio, United States; 3 Neurotrack Technologies, Redwood City, California, United States

## Abstract

Alzheimer’s disease (AD) is expected to triple by 2050, affecting 16 million Americans. As a result, it is essential to combat this alarming increase in cognitive impairment through early detection. Cardiometabolic risk factors have shown to be associated with higher risk of AD. The purpose of this study was to determine if cardiometabolic risk factors could predict executive function scores in a high-risk population. Fifty (60.9±8.8 years) high-risk adults (classified by the Australian National University Alzheimer’s Disease Risk Index) were enrolled in this study. Participants completed a 6-minute walking test, venous blood draw, blood pressure measurement, and the digit coding symbol test (DCS). Results were examined through a multiple linear regression with DCS as the dependent variable and age, sex, total cholesterol, high-density lipoprotein (HDL), low-density lipoprotein (LDL), glucose, 6-minute walking test, systolic blood pressure (SBP), and diastolic blood pressure (DBP) as predictor variables. The model explained 42% of the variance of DCS (p = .04) with SBP (45%; p = .003) as a significant predictor. LDL (p = .087) and DBP (p = .123) accounted for 24% and 22% of the variance for this model, respectively. These results suggest cardiometabolic risk factors predict executive function values in high-risk individuals. Higher SBP was significantly associated with lower DCS scores indicating SBP as a valuable tool for practitioners when evaluating cognitive decline. Further research should expand sample size and track values longitudinally to substantiate these claims.

